# Functional assessment of renal damage in children with primary vesicoureteral reflux

**DOI:** 10.3389/fped.2025.1429804

**Published:** 2025-04-10

**Authors:** Yaju Zhu, Yufeng Li, Jing Jin, Jiajia Ni

**Affiliations:** Department of Pediatric Nephrology, Rheumatology and Immunology, Xinhua Hospital Affiliated to Shanghai Jiaotong University School of Medicine, Shanghai, China

**Keywords:** vesicoureteral reflux, children, renal function, dimercaptosuccinic acid, effective renal plasma flow

## Abstract

**Objectives:**

To evaluate the renal function damage in children with primary vesicoureteral reflux (VUR).

**Methods:**

A total of 226 children with VUR (65 cases with left, 39 with right, and 122 cases with bilateral VUR) were screened. Eighty-five urinary tract infection (UTI) cases, without urinary malformations, during the same period were collected as controls. Age at diagnosis, body weight, renal ultrasound, VUR grade, serum creatinine level, dimercaptosuccinic acid (DMSA) level, and effective renal plasma flow (ERPF) values were retrospectively analyzed.

**Results:**

There were no significant differences in age at diagnosis between study groups. Total ERPF was significantly lower in the bilateral VUR group than in the control group. The ERPF in unilateral VUR was significantly lower than that in the contralateral or ipsilateral side in the control group (*P* < 0.001). The mean split renal function, as assessed by DMSA of VUR, was 28.00% and 29.12% on the left and right sides, respectively, both of which were lower than the control group's 40.27%. Renal damage was also correlated with a VUR grade (*P* = 0.008), a transverse diameter (*P* = 0.002), and pyelectasis (*P* = 0.037).

**Conclusion:**

Split renal function was impaired in the reflux kidney. The total ERPF in the bilateral VUR group was lower than that in the unilateral VUR group. Renal damage was correlated with a VUR grade, a transverse diameter, and pyelectasis.

## Introduction

Vesicoureteral reflux (VUR) is a congenital defect of the ureterovesical junction that leads to the retrograde passage of urine from the bladder towards the ureter and kidneys. The prevalence of VUR in children with febrile urinary tract infections (UTI) is approximately 30%, and this proportion is higher in neonates and small infants with UTI (1). VUR is one of the most common causes of UTI in children, and recurrent UTI may cause renal scarring and damage, as well as chronic kidney disease (CKD), including end-stage renal disease (ESRD) (2, 3). The formation of kidney scarring is positively correlated with reflux grade (4). VUR grade has a greater impact on renal function, but the extent of its impact has not been reported. Ultrasonography is an important imaging modality that has a positive correlation with renal function (5). Dimercaptosuccinic acid (DMSA) renal scintigraphy provides an accurate assessment of functional renal parenchyma (6). Dynamic renal scintigraphy provides an accurate assessment of split renal function in hydronephrosis (7). Technetium-99m-Ethylenedicysteine (^99m^Tc-EC) dynamic renal scintigraphy is beneficial for evaluating split renal function in transplant kidneys and percutaneous nephrolithotomy (8–10). ^99m^Tc-EC dynamic renal scintigraphy can predict the split renal function as an alternative to DMSA scintigraphy (11, 12).

This retrospective study aimed to evaluate the influence of VUR on renal function in children. In this study, we analyzed renal function by comparing kidney size, renal hypodysplasia, acquired renal scarring, and split renal function evaluated by nuclide imaging of the kidneys on both sides of the VUR and on the healthy side.

## Patients and methods

### Clinical data collection

This study was conducted as a retrospective chart review of pediatric patients evaluated at a single tertiary care center between January 2015 and December 2018. All children with febrile UTI or recurrent UTI were screened. Inclusion criteria is inpatient children, less than 14 years old. All patients finished voiding cystourethrography (VCUG), ultrasound, DMSA scanning and dynamic renal scintigraphy. Ultrasound, DMSA scanning and dynamic renal scintigraphy were performed within 3 months before or after VCUG procedure. Exclusion criteria is solitary kidney. The patients with missing DMSA scanning or dynamic renal scintigraphy data were also excluded. Patients with secondary VUR, including the posterior urethral valve, urethremphraxis, neurogenic bladder, duplex kidney, anal atresia, and tethered cord syndrome, were also excluded. Original records of all-grade VUR with UTI cases were reviewed. Children with UTI without urinary tract abnormalities during the same period were included as the control group. Urinary tract abnormalities in UTI cases were excluded by VCUG and ultrasound. Reflux on the left or right side was unilateral VUR, reflux on both left and right sides was bilateral VUR. Patient demographics (age at diagnosis, sex, and body weight), laboratory investigations (serum creatinine and serum cystatin C), imaging (ultrasound, scintigraphy, VUR grade), and clinical course were extracted. The ERPF values of split renal function were corrected by body surface area, which was calculated according to the following formula: body surface area = 0.035 × body weight (kg) + 0.1 m^2^ (13).

### Recurrent UTI

Recurrent UTIs were defined as at least three episodes of UTI in 12 months or at least two episodes in 6 months. All recurrent UTI cases had not received continuous antibiotic prophylaxis before VCUG.

### VUR grade

All cases of VUR were identified using VCUG. VUR was diagnosed by a demonstration of urine reflux into the upper urinary tract by conventional VCUG. VUR was graded according to the International Reflux Study Group classification: mild (grade 1–2), moderate (3), and severe (4–5).

### Renal parenchymal defects

Abnormal DMSA scan was defined as decreased uptake with loss of contours or cortical thinning, with distortion of parenchymal volume. All data files were re-evaluated at the coordinating center by the same nuclear medicine specialist using a workflow software (Philips, Amsterdam, Netherlands). Abnormal DMSA scan was characterized as focal (a single delimited area with decreased uptake), multifocal (more than one uptake defect), or generalized (a small kidney with generalized reduced tracer uptake).

### Dynamic renal scintigraphy

Dynamic renal scintigraphy were performed within 3 months before or after VCUG procedure. Patients were given 10–20 ml/Kg of water orally 30–40 min before the procedure. Posterior dynamic acquisition was performed after intravenous injection of 3.7 MBq/kg of body weight of 99mTc-ethylene-dicysteinc (99mTc-EC) and 1 mg/kg of body weight of furosemide (with a maximum of 20 mg). Images were processed by an independent senior nuclear medicine physician by a homemade software programmed with workflow software (Philips, Amsterdam, Netherlands). Regions of interest were manually drawn on kidneys, heart and C-shaped perirenal background. Relative function was determined using the Patlak-Rutland method, or the Area Under the Curve method in studies in which the cardiac curve did not meet enough quality, according to international consensus recommendation. Drainage was quantitatively assessed by NORA (normalized residual activity), Renal Output efficiency and Tmax. The operator then classified the drainage as normal, borderline or poor (14).

### Statistical analyses

Data are expressed as mean ± standard deviation. One-way way ANOVA, *t*-test, and Pearson *χ*^2^ tests were used to test the measurement data of the two groups. For continuous variables, Spearman's rank correlation coefficient was used to compare two variables, and the Mann–Whitney *U*-test was used between groups. Correlation of renal function were evaluated by using linear mixed models. Statistical analyses were performed using the SPSS software ver. 19.0 (SPSS Inc., Chicago, IL, USA). Statistical significance was set at two-tailed *P* < 0.05.

Ethical approval was obtained from the institutional ethics board of Xinhua Hospital, affiliated with the Shanghai Jiaotong University School of Medicine (Approval No. XHEC-D-2022-088).

## Results

### General information

In total, 226 children with VUR were screened. VUR was present in 348 kidneys, bilaterally in 122 cases, left-sided in 65 cases, and right-sided in 39 cases. The mean age at diagnosis in the bilateral VUR, unilateral VUR, and UTI control group was 21.65 ± 27.99 months, 28.35 ± 35.79 months, and 30.45 ± 31.72 months, respectively. The average age at diagnosis and body weight showed no significant differences among the groups. The male ratio was much higher in the VUR group than in the UTI control group (*P* = 0.037). Recurrent UTIs were more likely to occur in children with bilateral reflux (*P* = 0.048) (Table 1).

**Table 1:** Total renal function in VUR.

Variable	Unilateral VUR (*n* = 104 cases)	Bilaterally VUR (*n* = 122 cases)	UTI control (*n* = 85 cases)	*F* value	*P* value
Age at diagnosis (month)	28.35 ± 35.79	21.65 ± 27.99	30.45 ± 31.72	2.19	0.114
Body weight (Kg)	13.71 ± 9.38	12.27 ± 7.61	13.93 ± 8.80	1.273	0.281
Boys (*n*, %)	66, 63.46%	75, 61.48%	31, 36.47%	6.605	0.037
Creatinine (umol/L)	25.19 ± 13.81	26.72 ± 17.84	22.87 ± 10.90	1.659	0.192
Cystatin C (mg/L)	0.54 ± 0.50	0.60 ± 0.57	0.66 ± 0.37	1.388	0.251
Recurrent UTI (*n*, %)	67, 64.42%	92, 75.41%	51, 60.00%	6.09	0.048
Dynamic renal scintigraphy					
Total ERPF	219.74 ± 130.46	207.36 ± 110.81	287.56 ± 76.16	14.499	<0.001

VUR, Vesicoureteral reflux; UTI, Urinary tract infection; ERPF, effective renal plasma flow

### Total renal function in VUR

The serum creatinine values in unilateral VUR, bilateral VUR, and control groups were 25.19 ± 13.81 µmol/L, 26.72 ± 17.84 µmol/L, and 22.87 ± 10.90 µmol/L, respectively. The serum cystatin C values in unilateral VUR, bilateral VUR, and control groups were 0.54 ± 0.50 mg/L, 0.60 ± 0.57 mg/L, and 0.66 ± 0.37 mg/L, respectively. Serum creatinine and cystatin C levels were not significantly different between the groups (*P* = 0.192 and *P* = 0.251, respectively) (Table 1).

**Figure 1:**
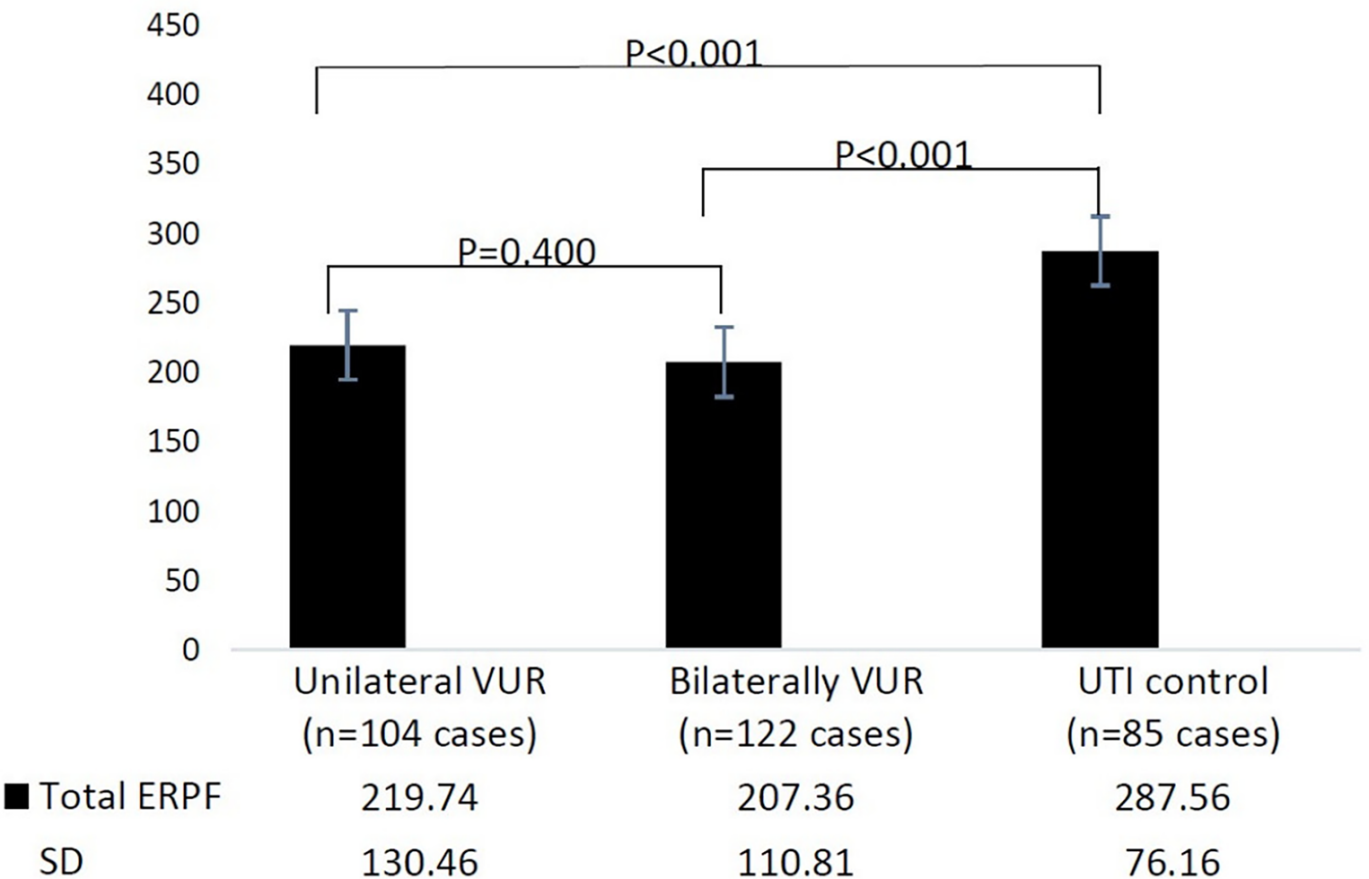
Total effective renal plasma flow (ERPF) in different groups. UTI, urinary tract infection; VUR, vesicoureteral reflux; ERPF, effective renal plasma flow.

Total effective renal plasma flow (ERPF) values of unilateral VUR, bilateral VUR, and the control group were 219.74 ± 130.46 ml/min/m^2^, 207.36 ± 110.81 ml/min/m^2^, and 287.56 ± 76.16 ml/min/m^2^, respectively. The total ERPF values in the unilateral VUR and bilateral VUR groups were significantly lower than those in the control group (*P* < 0.001 and *P* < 0.001, respectively). There was no significant difference in the total ERPF value between the unilateral and bilateral VUR groups (*P* = 0.400) (Figure 1).

### Split renal function in VUR

Reflux occurred in 348 kidneys, and the 104 contralateral, unaffected-side kidneys included 65 left non-reflux kidneys and 39 right non-reflux kidneys. DMSA scans showed split renal function in unilateral reflux kidneys, contralateral unaffected-side kidneys, and ipsilateral side kidneys in the control group, which were 31.68% ± 25.94%, 42.02% ± 29.06%, and 40.00% ± 20.35%, respectively. Split renal function in unilateral reflux kidneys was much lower than that in ipsilateral kidneys in the control (*P* < 0.001) and contralateral unaffected kidneys (*P* < 0.001). Split renal function was not significantly different between the contralateral unaffected-side kidneys and ipsilateral side kidneys in the control group (*P* = 0.518) (Table 2).

**Table 2:** Split renal function in VUR and impact on split renal function.

Variable	Unilateral reflux kidneys (*n* = 348)	Contralateral un-affected kidneys (*n* = 104)	Ipsilateral side kidneys in control (*n* = 170)	*F* value	*P* value
Age at diagnosis (month)	23.65 ± 30.59	28.35 ± 35.79	30.45 ± 31.62	2.87	0.057
Body weight (Kg)	12.70 ± 8.18	13.71 ± 9.38	13.93 ± 8.77	1.411	0.245
Creatinine (umol/L)	26.26 ± 16.71	25.19 ± 13.81	22.87 ± 10.87	2.98	0.052
Cystatin C (mg/L)	0.58 ± 0.55	0.54 ± 0.50	0.66 ± 0.37	2.398	0.092
Recurrent UTI (*n*, %)	251, 72.12%	67, 64.42%	102, 60.00%	153.66	<0.001
VCUG					
VUR grade	3.49 ± 1.16	0	0	NA	NA
Ultrasound					
Length diameter (mm)	56.85 ± 25.01	58.61 ± 28.21	68.47 ± 13.36	14.837	<0.001
Transverse diameter (mm)	23.21 ± 10.85	23.51 ± 11.53	26.51 ± 5.17	6.818	0.001
Pyelectasis (mm)	4.15 ± 6.34	1.06 ± 2.93	0.37 ± 1.43	39.39	<0.001
Dilatation of ureters (mm)	1.14 ± 2.89	0.08 ± 0.78	0.04 ± 0.54	18.745	<0.001
DMSA					
Split renal function (%)	31.68 ± 25.94	42.02 ± 29.06	40.00 ± 20.35	10.246	<0.001
Abnormal DMSA scan					
Normal	65, 18.68%	63, 60.58%	54, 31.76%	77.218	<0.001
Focal	93, 26.72%	24, 23.08%	72, 42.35%		
Multifocal	95, 27.30%	3, 2.88%	36, 21.28%		
Generalized	49, 14.08%	0, 0.00%	2, 1.18%		
NA	46, 13.22%	14, 13.46%	6, 3.53%		
Dynamic renal scintigraphy					
ERPF	102.58 ± 70.04	134.00 ± 81.51	143.78 ± 40.06	25.8	<0.001

mm, millimeter; UTI, Urinary tract infection; VCUG, Voiding cystourethrography; VUR, Vesicoureteral reflux; DMSA, dimercaptosuccinic acid; ERPF, effective renal plasma flow; focal renal scarring: single delimited area with decreased uptake; multifocal renal scarring: more than one uptake defect; generalized renal scarring: a small kidney with generalized reduced tracer uptake; NA, no data.

The ERPF value of the split renal function in unilateral reflux kidneys and contralateral unaffected-side kidneys were 102.58 ± 70.04 ml/min/m^2^ and 134.00 ± 81.51 ml/min/m^2^, respectively. The ERPF values in reflux kidneys were much lower than those on the contralateral unaffected-side kidneys and the ipsilateral side kidneys in the control group (*P* < 0.001 and *P* < 0.001, respectively). In contrast, ERPF values in the contralateral kidney of reflux were not significantly different from those on the ipsilateral side in the control group (*P* = 0.231) (Table 2).

### Impact on split renal function

The length diameters of the reflux kidneys and contralateral unaffected kidneys were 56.85 ± 25.01 mm and 58.61 ± 28.21 mm, respectively. Both length diameters in reflux and contralateral unaffected kidneys were much shorter than those in ipsilateral kidneys in the control group (*P* < 0.001 and 0.001, respectively). The transverse diameters of reflux and contralateral unaffected kidneys were 23.21 ± 10.85 mm and 23.51 ± 11.53 mm, respectively. Both transverse diameters in the reflux and contralateral un-reflux kidneys were much shorter than those in the ipsilateral kidney in the control group (*P* < 0.001 and 0.014, respectively). The pyelectasis of reflux and contralateral unaffected kidneys were 4.15 ± 6.34 mm and 1.06 ± 2.93 mm, respectively. Both pyelectasis in reflux kidneys were much larger than those in contralateral unaffected kidneys and ipsilateral kidneys in the control group (*P* < 0.001 and 0.001, respectively). There was no significant difference between the contralateral unaffected kidneys and ipsilateral kidneys in the control group (*P* = 0.266). The dilatation of ureters of reflux kidneys and contralateral unaffected kidneys were 1.14 ± 2.89 mm and 0.08 ± 0.78 mm, respectively. Both dilatation of ureters in reflux kidneys were much larger than those in contralateral unaffected kidneys and ipsilateral side kidneys in the control group (*P* < 0.001 and <0.001, respectively). There was no significant difference between the contralateral unaffected kidneys and ipsilateral kidneys in the control group (*P* = 0.896) (Table 2).

Abnormal DMSA scan detected in the reflux, contralateral unaffected, and ipsilateral kidneys in the control group were 68.1%, 25.96%, and 64.81%, respectively. A small kidney with generalized reduced tracer uptake was more common in reflux kidneys (*P* < 0.001) (Table 2).

Using linear mixed models, we tested the following baseline variables for the prediction of renal damage: age at diagnosis, body weight, type of presentation, length diameter of the kidney, transverse diameters of the kidney, pyelectasis, dilatation of ureters, VUR grade, ERPF, febrile UTI, and recurrent UTI before inclusion. Renal damage was also correlated with a VUR grade (*P* = 0.008), a transverse diameter (*P* = 0.002), and pyelectasis (*P* = 0.037).

## Discussion

VUR is a condition in which urine flows back from the bladder to the ureter or pelvis. VUR can be classified as primary or secondary according to its etiology. The enrolled cases in this study were all primary VUR cases, mainly caused by abnormal development of the vesicoureteral flap, which is closely related to genes (15). VCUG is the “gold standard” technique for detecting VUR. It provides high-resolution anatomical images of the renal parenchyma, calyx, pelvis, and bladder. The ureters and urethra can be partially visualized. VUR was divided into 1–5 grades according to the degree of urine reflux. Higher VUR grades had a greater probability of renal dysplasia or scarring formation and a greater chance of urinary tract infection (2). Recurrent urinary tract infections can easily cause renal scarring, proteinuria, hypertension, and other symptoms of reflux nephropathy. Severe reflux, bilateral reflux with renal scarring, hypertension, proteinuria, and decreased GFR are risk factors for the progression to CKD or ESRD (13). The early assessment of renal function in children with VUR is conducive to early intervention and improved prognosis.

This study included 104 children with unilateral VUR, and the mean age at diagnosis was not significantly different from that of the control group. The diameter of the reflux kidney was smaller than that of the control group, indicating that kidney development in the reflux kidney was significantly affected. The renal ERPF value in the reflux kidney was significantly lower than that in the contralateral unaffected and ipsilateral kidneys in the control group. Split renal function in reflux kidneys was significantly impaired. The split renal ERPF value of the bilateral reflux kidneys was lower than that of the same side in the control group, and the total ERPF value of the bilateral VUR was significantly lower than that of the control group. Patients with bilateral VUR have obvious renal impairment, which should be strengthened during long-term follow-up monitoring.

^99m^Tc-DMSA is the gold standard for diagnosing renal scarring. It is commonly used to measure split-renal function. Split renal function is considered to range from 45% to 55% of the total uptake in healthy kidneys (16). It may be difficult to accurately evaluate lesions in bilateral VUR because the relative uptake remains stable. ^99m^Tc-EC dynamic renal scintigraphy is beneficial for evaluating transplant kidney function (8). Following intravenous administration of ^99m^Tc-EC, some (17%) of it is filtered in the glomeruli, while a major portion (50%) is secreted in the proximal part of the tubules by organic anion transporters (16). ERPF correlates with eGFR (17). ^99m^Tc-EC was used to evaluate split renal function in hydronephrosis or UTI (18). The unilateral renal ERPF value of bilateral VUR was lower than that of the same side in the control group, and the total ERPF value of bilateral VUR was significantly lower than that of the control group.

The predictive factors for deterioration were recurrent febrile urinary tract infections, bilateral abnormalities, and reduced total glomerular filtration rate. Deteriorated renal status was more common in cases diagnosed prenatally than in those detected after urinary tract infection (19, 20). In this study, the predictive factors for renal function deterioration were analyzed. Abnormal DMSA scan (*P* = 0.003), VUR grade (*P* = 0.008), transverse diameter (*P* = 0.002), and pyelectasis (*P* = 0.037) were significantly correlated with renal function damage. Severe VUR was associated with impaired renal function (13). Renal parenchymal defects were observed in 87% of children at baseline, with a strong correlation with renal function, which is in accordance with several previous reports on congenital renal dysplasia (4, 6, 7, 21). VUR is often associated with recurrent urinary tract infections, which can lead to scarring and impaired kidney function. The average reflux was above grade 3, indicating moderate and severe reflux. Moderate and severe reflux are often accompanied by renal pelvis and ureteral dilation. Therefore, the degree of pyelectasis and ureteral dilation on the reflux side were higher than those in the control group.

The study screened 348 VUR kidneys at a tertiary center, and the outcomes were objectively measured by professional physicians. Nevertheless, owing to the retrospective design of the study, we cannot make a causal conclusion. Additionally, selection bias for a single-center study with measurement bias might not be excluded. It is possible that residual confounders, such as socioeconomic factors, which might introduce study bias. Due to the short term follow up and single-center study design, the generalization of our conclusions might be limited. Our findings warrant further study with the need for a well-designed, large-scale, long term follow up, prospective study.

In this retrospective study, we analyzed the effect on renal function in primary vesicoureteral reflux children. Split renal function in the reflux kidney was impaired. The total ERPF in the bilateral VUR group was lower than that in the unilateral VUR group. Renal damage was correlated with a VUR grade, a transverse diameter, and pyelectasis. Bilateral VUR with high VUR grade, more renal scarring and hydronephrosis requires attention to strengthen follow-up.

## Data Availability

The data analyzed in this study is subject to the following licenses/restrictions: The Ethical Review is needed before using the dataset. Requests to access these datasets should be directed to liyufeng@xinhuamed.com.cn
